# Omental Torsion after Laparoscopic Roux-en-Y Gastric Bypass Mimicking Appendicitis: A Case Report and Review of the Literature

**DOI:** 10.1155/2016/7985795

**Published:** 2016-02-25

**Authors:** Alexandre Descloux, Giacinto Basilicata, Antonio Nocito

**Affiliations:** Department of Surgery, Kantonsspital Baden, Dättwil, 5404 Baden, Switzerland

## Abstract

*Introduction*. Laparoscopic Roux-en-Y gastric bypass (LRYGBP) is a common procedure in obesity surgery. The aim of an antecolic approach is to reduce the rate of internal herniation. Our aim is to make bariatric surgeons aware of another possible complication of antecolic LRYGBP.* Methods and Results*. We present a case report of omental torsion 24 months after antecolic LRYGBP presenting as an acute abdomen, suggesting appendicitis. During diagnostic laparoscopy, omental infarction due to torsion was observed. Resection of the avital omentum was performed.* Discussion*. Omental torsion after antecolic LRYGBP is a rare complication. When appearing in the early postoperative phase, it may mimic an anastomotic leakage. It may also occur as late complication, presenting with acute abdomen as an appendicitis.

## 1. Introduction

Laparoscopic Roux-en-Y gastric bypass (LRYGBP) is a common procedure in obesity surgery. One aim of an antecolic approach is to reduce the rate of internal herniation [1]. This case report should make us aware of another possible complication due to this approach.

## 2. Methods and Results

We performed an antecolic LRYGBP on a 31-year-old female patient with a body mass index of 50.3 kg/m^2^. During surgery, the great omentum was divided in its length to reduce tension on the gastrojejunal anastomosis.

**Figure 1:**
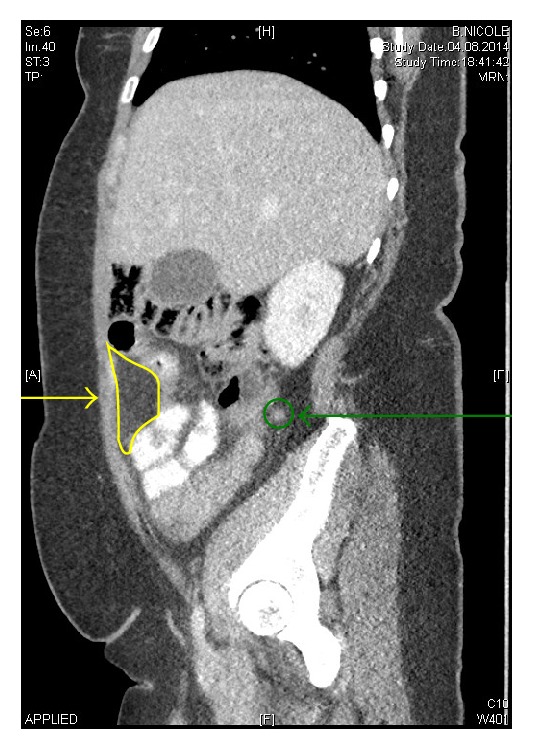
Preoperative CT scan, sagital view; infarcted omentum (yellow line and arrow), appendix vermicularis (green circle and arrow).

24 months later, the patient presented to our emergency room with lower quadrant abdominal pain. Fever was not observed. Her body mass index was 37.7 kg/m^2^. The palpation at the McBurney's point was painful with rebound tenderness. White blood cell count was not elevated, and C-reactive protein (CRP) was moderately high (42 mg/L). Other routine abdominal laboratory findings were unremarkable. Sonography showed nothing in particular. The patient was discharged on patient's demand and came back the following morning for clinical reevaluation. She presented with progressive pain and an elevation of the CRP (91 mg/L). A computer tomography was performed. The appendix vermicularis showed a diameter of 10 millimeters (Figure 1). The patient consented to diagnostic laparoscopy.

**Figure 2:**
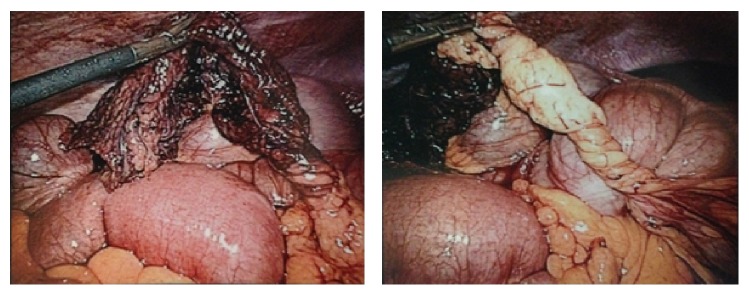
Intraoperative situs demonstrating omental torsion and infarction.

During laparoscopy, omental infarction due to a torsion of the right half of the divided great omentum was observed (Figure 2). Resection of the avital omentum was performed. The slightly enlarged appendix vermicularis was resected as well. Histological findings showed an infarction of the omentum without pathologies of the appendix. On postoperative day one, the patient was asymptomatic and was discharged on day two.

## 3. Discussion

The first case of omental torsion after antecolic gastric bypass was observed by Dallal and Bailey 2006 [2]. They reported even three cases. All cases presented acute abdominal pain at day three after antecolic LRYGBP, requiring emergent laparoscopy for diagnosis. Similar to our observation, Bestman et al. published in 2009 an isolated case months after surgery [3].

Omental torsion after antecolic LRYGBP is a rare complication. When appearing in the early postoperative phase, it may mimic an anastomotic leakage. Its happening as late complication may illustrate an acute abdomen like an appendicitis. Conservative management after accurate diagnosis is also an option [4].

Reviewing the current literature, we believe to report only the sixth case of omental torsion following gastric bypass procedure.
